# Perceptions of portfolio assessment in family medicine graduates: a qualitative interview study

**DOI:** 10.1186/s12909-022-03991-7

**Published:** 2022-12-30

**Authors:** Faten A. AlRadini

**Affiliations:** Department of Clinical Sciences, College of Medicine, Princess Nourah bint Abdulrahman University, 11671 Riyadh, Saudi Arabia

**Keywords:** Programmatic assessment, Portfolio assessment, Perceptions, Qualitative, Reflection, Mentor, Professional development

## Abstract

**Background:**

The use of the portfolio methodology in medical education can serve as a tool for learning, assessment, and reflection on practice. This study concentrates on perceptions of the portfolio assessment methodology among participants in the Saudi Diploma of Family Medicine program.

**Methods:**

In this qualitative interview study, data were collected and analysed using a grounded theory approach.

**Results:**

Nine codes emerged: (1) Importance of understanding the definition, objectives, and process of portfolio assessment, (2) Impact of different understandings on the part of various trainers, (3) Role of the type of assessment, (4) Workload and stress of portfolio assessment, (5) Effectiveness of the portfolio contents, (6) Role of the mentor’s feedback, (7) Role in the learning process, (8) Role in practice, (9) Suggestions for portfolio improvement. Open codes were then regrouped into three axial codes: context, strategy, and outcome of portfolio assessment.

**Conclusion:**

This study explored a general explanation of portfolio assessment shaped by the postgraduate students. It identifies the importance of portfolio understanding in student acceptability of the portfolio assessment methodology. Thus, proper implementation is vital for the success of assessing the student by the portfolio methodology. The students perceived reflection as the most valuable part of the process, which facilitated their learning, confidence, and self-assessment. Mentor feedback is a good strategy for coping with portfolio challenges. Our findings provide some evidence of positive outcomes of portfolio assessment in practice and professional development.

**Supplementary Information:**

The online version contains supplementary material available at 10.1186/s12909-022-03991-7.

## Background

Postgraduate medical education (PGME) is the continuation of medical study and the introduction to a medical speciality, after the achievement of a professional degree in undergraduate medical education [1]. Therefore, a focus on professional development and competence in postgraduate medical education is crucial. Medical competence is a combination of knowledge, skills, problem solving ability and attitude. Programmatic assessment is established to assess competence in PGME. It is not a method in itself but a conceptual assessment design model that is based on four principles: use of multiple instruments with triangulation of information, longitudinal, multiple quality approach and meaningfulness. Programmatic assessment minimises arbitrary decisions about student level of competence through various assessment methods throughout the year. It supports the student with meaningful feedback to be a self-directed learner [2].

Therefore, in any teaching or training program of PGME, self-assessment of the student’s professional competence is encouraged [3]. The ideal assessment method of learners’ ability in self-assessment and reflection, which are key components of professional development, is the portfolio [3]. ”A portfolio may be described as a collection of evidence maintained and presented for a specific purpose” [4]. A portfolio can assess some learning outcomes which cannot be measured by other assessment methods, such as reflection, self-directed learning, self-assessment and professionalism [3, 5]. It can assess the in-depth profile of student performance [3]. Portfolios in medical education can be used as a learning method to achieve learning goals, as a tool for reflecting on practice, and as a formative or summative assessment of learning [6]. Portfolios have a wide range of uses in PGME such as recertification, revalidation and continuous professional development [6]. A portfolio is a good tool for reflecting critically on learning and for polishing the reflective skills required to succeed in a career [7, 8].

Although most studies have concluded that the portfolio methodology is valuable, two studies found that the role of portfolios in learning support was questionable as they may require excessive paperwork which could interrupt proper clinical learning, thereby increasing student anxiety and frustration [4, 9] The later study also found that attitudes toward portfolios improved after paperwork was reduced [9]. A student’s experience with portfolios may initially be negative because compiling a portfolio might be time-consuming, requiring considerable effort to record impressions and collect documents [7, 9]. The process might also be challenging even for senior staff to become familiar with the review and assessment of portfolios [9]. However, some students perceive these difficulties as a valuable experience if supported by continuous reading and reflection [7]. Portfolios can be augmented by regular feedback or mentoring sessions [4]. Evidence has shown that faculty support is frequently required to support the successful implementation of portfolios [3, 6]. One study examined student attitudes in a longitudinal portfolio mentoring program using tasks of written reflection to increase reflective competence [10]. The study revealed that negative attitudes toward reflection were due to misunderstanding, insufficient knowledge and uncertainties. It found that mentor-mentee relationships can influence student attitudes toward portfolio assessment, resulting in either positive or negative changes throughout the mentorship journey.

Although portfolios can be a valid and reliable method of assessment in some studies [4], it is advisable to triangulate portfolio data with other methods in a summative assessment [6]. Tochel et al. found that portfolios might not be sufficient as a method of summative assessment but could be useful in qualitative personal development [6]. Therefore, a model of programmatic assessment has been introduced that triangulates different assessment methods with longitudinal mentorship using the portfolio throughout the duration of the program [11]. This type of assessment fits work-based learning such as PGME as it can measure the top in Millers’ pyramid (The “Does” peak, when performance is integrated into practice) [12]. Programmatic assessment is theoretically promising, but empirical information about its extent and suitability to various educational contexts are still matters of ongoing research [2]. Therefore, the aim of this study was to explore the process of portfolio assessment in a postgraduate Family Medicine context and investigate the impact of the portfolio methodology in future practice and professional development.

The Saudi Diploma of Family Medicine (SDFM) offers a new experience of formative assessment of student performance by the portfolio methodology in Saudi Arabia. Students are the primary target of learning, and their perspectives are of great value to identify the strengths, weaknesses, challenges and areas for improvement. The method reflects the use of a Kirkpatrick level one training evaluation [13].

## Methods

### Study context

The study was conducted in the Postgraduate Centre of Family Medicine at Riyadh, Saudi Arabia, where the SDFM program is conducted. It was carried out in a natural setting to provide a holistic understanding of the experience [14]. The SDFM is a postgraduate training program under the umbrella of the Ministry Of Health (MOH) in Saudi Arabia. It was established to compensate for the critical shortage of family physicians in primary health care centres in MOH, Saudi Arabia. The SDFM is completed after 14 months of training in hospitals and primary care centres.

This program has ten to thirteen trainees in each year of training. The program consists of a family medicine rotation that is conducted in primary health care centres, and different medical specialty rotations which are run in hospital placements. Before portfolio implementation, an orientation lecture about portfolio assessment is held for all trainers in the SDFM. Subsequent meetings are conducted by the program director to discuss the changes and updates of portfolio assessment with all the trainers. In the introductory course of the SDFM program, a lecture on portfolios is conducted for all the trainees and all the trainers are invited. After that, the trainees are appointed to mentors for portfolio follow up and assessment, who are family medicine consultants and full-time trainers in the SDFM. Mentor meetings should be conducted monthly or at the end of the rotations, unless the trainee or the mentor has other commitments that require a meeting to be rescheduled. The current SDFM portfolio consists of a lever arch file containing a record of various educational and practical evidence, along with a variety of assessment tools, as shown in Table 1. These assessment forms are completed by family medicine trainers in the family medicine rotations and clinics. In the hospital placements, they are usually completed by non-family medicine clinical supervisors in a particular rotation.

**Table 1:** Assessment tools in the Saudi Diploma of Family Medicine portfolio

	Assessment tool	Frequency
1	Educational activity evaluation form by peer	1–2/ year
2	Educational activity evaluation form by supervisor	1–2/ year
3	Case-Based Discussion or clinical case summary	7 cases/ month
4	Clinical Skills	8 skills/ month
5	Direct Observation of Procedural Skills (DOPS)	8 skills/ month
6	Clinical logbook Diary	18 cases/ month
7	Mini Clinical Evaluation Exercise (Mini-CEX)	Once/ month
8	Reflective Learning	1–2/ month
9	End of Rotation Evaluation form	1–2/ month
10	Report of mentor meeting for portfolio assessment on five scales with mentor’s comments	Once/ month

### Study design

In this qualitative research, a grounded theory approach is used to understand the process of portfolio assessment in the SDFM program. Grounded theory is a research methodology that differs from an existing theoretical framework in that they are considered “grounded” in the participants’ explanations or interpretations. It is an inductive approach that is used to increase the understanding of social phenomena. The grounded theory was initially developed by Glaser and Strauss in the 1960s [15]. It is a general methodology for advancing theory arising from systematic data gathering and analysis [16]. The developing theory emerges from ongoing data analysis and can inform the process of further data collection. There are four main criteria: fitness, understanding, generality and control [17]. Fitness represents the correspondence of the theory with the data. Understanding requires that the theory is comprehensible to those involved. Generality requires that the theory is applicable in a variety of contexts. Control indicates that the theory offers control regarding action toward the phenomenon.

Grounded theory has several important advantages for this study compared to other qualitative research methods [15]. It is not only a paradigm but also a unified and systematic method of analysis. It includes methods for validating studies and integrates well with other approaches. Grounded theory features extensively in the literature, and has been used in qualitative research for over 50 years [16], meaning that it is well tested and challenges have been thoroughly explored.

### Sampling and recruitment

In this study, all participants were graduates of the postgraduate SDFM program. Opportunistic sampling was used to recruit the participants from different years and different genders to explore a range of views about portfolio assessment [18]. Inclusion criteria considered whether graduates had completed the SDFM program and had experience with portfolio assessment. Current Students or former graduates without experience in portfolio assessment were excluded. An email invitation was sent to all those who had taken part in the program over the last three years (32 participants).

### Data collection

In-depth face-to-face, audio-recorded, semi-structured interviews were conducted that lasted between approximately 45 and 60 min. Open-ended questions were used to evoke an in-depth description of participants’ perceptions of portfolio assessment in the SDFM program, as shown in the interview guide (Appendix 1). The interviews started with the questions from the interview guide. Then, clarifying questions were added in subsequent interviews as new themes arose during the first and second interviews.

### Data analysis

The analysis was based on the principles of grounded theory. An important feature of the grounded theory method involves systematic methods of data collection and analysis. These methods are described by Strauss and Corbin [16] and are summarised below.

1. Selecting the research question
2. Acquiring the data
3. Coding

All the interviews were transcribed and analysed. The transcripts were printed and hand-coded. Initial Codes were developed after iterative readings of all transcripts, and all emerged from reading the data. All the codes and corresponding sections of the texts were incorporated into an Excel document with the anonymous identifier for each person interviewed. One entire transcript was re-coded independently by an expert in qualitative research to ensure consistency and increase coding reliability. Fortunately, there was excellent agreement and the other transcripts were reviewed in light of this coding. Further discussion with the expert resulted in agreement of nine open codes. Then, axial coding was conducted as a second phase in the analysis. Axial coding relates the categories, which emerged from the open codes, with subcategories. It organises and synthesises the initial codes to be coherent and characteristics of the process being explored in the grounded theory, which is here portfolio assessment. It is used to construct a focused frame of research application.

## Results

Only seven positive responses were obtained, and all were included in the analysis of the study. Fortunately, these were distributed between the different years, three from the first year, two from the second year and two from the third year and included both males and females. The last interview did not add any new information despite adding extra questions to clarify the issues from the previous interviews, which might indicate data saturation [19].

Nine codes emerged from the data in respect to participants’ perceptions of portfolio assessment in the SDFM program.

- 1. Importance of understanding the definition, objectives and the process of portfolio assessment

Portfolio assessment was a challenging experience for most of the SDFM participants. All of them faced many difficulties, particularly at the beginning of the process, which they attributed to an incomplete understanding of the assessment. They all agreed that portfolio assessment was a useful and beneficial experience, but they linked its effectiveness to their understanding of its definition, objectives, background and the process. Specific comments of participants are included as italicised insets.

*The biggest challenge was at the beginning, as the portfolio wasn’t fairly explained and we didn’t comprehend its meaning so we were worried we might never get it. However, as time passed and with the mentors’ explanation, we could finally get it.*

At the SDFM, there was an introductory lecture about portfolio assessment but according to some participants, it was not effective in clarifying the confusion. They believed that this could have been due to the fact that they were new to the experience of portfolio assessment, the timing of the lecture was wrong, its structure was poor, or it might just be necessary to provide further clarification or discussion about portfolio assessment.

Different understandings of portfolio assessment in SDFM were noted mainly regarding the reflection aspect. Participant understanding of the portfolio contents differed and this was a concern for most of the participants, until they practised using the portfolio and received guidance from their mentors. After that, the participants came to appreciate the effectiveness of the portfolio once they were able to understand it, which was achieved later in the program.

- 2. Impact of different understandings on the part of various trainers

Different understandings of portfolio assessment among various trainers were identified by all the participants. This confusion was one of the factors that led to misunderstandings of the assessments.

*Actually the experience of the portfolio is new. Even after it was explained to us, the requirements were still different for mentors. There was no conformity in the picture; it was really confusing and unclear.*

Different understandings were greater among the clinical trainers and consultants in other specialties but were also clearly mentioned among family medicine trainers and mentors. The assessment tools were used in different ways by mentors after a period in the program which may have added to the confusion among the mentors, too.

*After I organised it a certain way, my mentor asked me to change it after 3 or 4 rotations, so I had to exert extra effort and time.*

According to some participants there was an introductory lecture about portfolio assessment which was conducted for the trainees; however only a few trainers attended, unfortunately.

- 3. Role of type of assessment

The type of portfolio assessment and whether it was formative or summative was not clear for some of the participants until late in the program.

*I am frankly shocked that the portfolio evaluation is not summative because I expected it to be evaluated by marks. I’m sure if it had such marks, it would be great because it contains a lot of effort in writing and doing what is supposed to be done.*

Some participants claim the type of formative assessment as the main reason for trainee dissatisfaction with portfolio assessment in the SDFM program.

*There was dissatisfaction because of the quantity of papers and because it was new and not credited so it was like an extra effort with no pay back or return.*

Trainee perceptions of the effectiveness of the assessments varied widely. Most participants were supportive of a summative evaluation, while some would prefer a mixture of summative and formative while one participant would prefer formative assessment alone. Some of the participants considered portfolio assessment to be a fair assessment method because it was longitudinal and measured cumulative student performance, unlike other methods, which depend on knowledge at a particular time under specific conditions, such as multiple-choice questions or an objective structured clinical examination.

- 4. Workload and stress of portfolio assessment

One important perception that all participants shared was the workload and time demands of portfolio assessment. Stress accompanied the workload of most of the participants. Some participants were overcome with physical stress, some with psychological stress and a minority reported financial stress. Paperwork and time barriers were the main reasons for trainee dissatisfaction. All the participants agreed that completing the portfolio was a time-consuming task: selecting the cases, writing them up, editing and organising the portfolio. One participant mentioned the economic aspect of the portfolio, but this was not a concern for the other participants.

*…frankly there are a lot of papers I need to fill at the end of each rotation, so it obstructed me and took a lot of my time to the point that it sometimes took me a week to organise the portfolio* [sic].

The frequency of the required numbers of each assessment tool played a major role in trainees’ acceptability. Most of the participants perceived the logbook as the worst requirement because it was required more frequently. On the other hand, reflection and Mini-CEX were the most acceptable and useful as they took less time. Stress caused by the portfolio was higher during the hospital rotations than in the family medicine setting. As the trainees proceeded in the program, most of them succeeded in controlling the stress caused by the portfolio work. However, stress was continuous for some participants. The mentor was an important factor affecting participant responses to the stress.

*…became less irritated because some of us ended up appreciating the portfolio and some others managed to cope with it and eventually comprehended its point. I believe those students who remained irritated until the very end were improperly instructed by the mentor.*

Only one participant perceived no stress, but that participant had previous experience with a portfolio project in undergraduate medical education.

- 5. Effectiveness of the portfolio contents

The participants’ perceptions regarding the effectiveness of the portfolio contents varied widely in respect to different assessment tools.

*As for the short cases (log book), I do not find them useful, and I think that it is a burden more than it’s a gain.*

Regarding the skills and DOPS form, its effectiveness varied among the participants. Some of the participants found it useful as a motive for the achievement of practical competency under supervision of the consultant. Others did not find it useful as they questioned its applicability in the family medicine setting. They saw it as an obstacle, particularly in special hospital rotations. They suggested specifying the skills and DOPS for rotations that require practical competency such as surgery and obstetric rotations.

All the participants perceived the effectiveness of case-based discussion and Mini-CEX as supportive tools for improving their clinical skills. It helped them to assess their strengths and weaknesses. Reflection was considered the best tool among all the participants except one who appreciated the Mini-CEX more. Reflection encouraged self-assessment and self-directed learning. It also supported broad thinking in respect to knowledge, skills and behavioural responses to medical problems. One of the participants admired reflection as a means of psychological support in such an interdisciplinary program that is full of stress.

*I believe the reflection part was important in respect to the psychological aspect because we would sometimes feel down because of some attitudes and situations we encountered during training in other specialties.*

It is noted that the participants were highly appreciative of the assessment tools which contain trainers’ comments or feedback and marginalised the tools with no trainer discussions or feedback. However, one participant commented:

*Personally, I believe all the portfolio content was useful, including the short cases, long ones and the Mini-CEX*.

- 6. Role of the mentor’s feedback

Mentor feedback was appreciated by all participants as a crucial component of portfolio assessment.

*The meeting with the mentor to discuss the portfolio was an important addition to the portfolio since it was the thing we benefited most throughout the program. It is true that there can be a meeting with the mentor without the portfolio…but portfolio sets our dialogue and problems…the effectiveness of the portfolio might decrease without the mentor* [sic].

Most participants preferred frequent meetings with their mentor as a supportive tool for trainee achievements. However, one participant found it stressful, even as a formative assessment, and would have preferred it to be frequent only at the beginning of the program, then less frequent, but this participant still believed in the importance of feedback. Mentors play a major role in portfolio acceptability as some trainees did not appreciate the value of the portfolio until the end of the program. Mentor feedback in portfolio assessment was perceived as supportive for all the participants throughout their training.

*Most of the dissatisfied trainees were with certain mentors. The mentor and his understanding had great effect on the effectiveness of the portfolio, and on the degree of the trainee’s acceptance to the portfolio* [sic].

- 7. Role of portfolio assessment in the learning process

Portfolio assessment helped all the participants in assessing their performance throughout the program by identifying their strengths and weaknesses. The type of assessment tool plays a major role in recognizing the impact of the portfolio in the learning process, as one participant commented:

*First it helped me to understand the strengths and weaknesses along with the guidance to improve myself, which I really did in the areas of (reflective learning) and (Mini CEX).The second part was one that didn’t add much to me but yet it wasn’t an obstacle… like the DOPS, it didn’t add to me anything but at the same time it wasn’t much of an obstacle, as the required number was little. Third, there were also things that took me so much effort and have been obstacles for me, like the logbook. There were so many cases to write with many things to repeat which did not add to me but held me back and took me much time* [sic].

Some participants found it useful before exams to review the detailed clinical cases that were supported by scientific discussion or medical guidelines or updates. Others found they did not need to go back to the portfolio as they found writing up the cases enabled them to memorise the knowledge without going back to read it again. It was also a good motivator for the students to appreciate their achievements and significantly promoted their self-confidence.

*The portfolio was a means of follow up and constant activity... It boosted me to achieve my learning objectives. I felt very proud and confident at the end of the diploma program when I went through my achievements in the portfolio. It is important to document achievement and success for the sake of more success.*

One participant highlighted the portfolio’s role in gaining searching skills. Another appreciated the portfolio’s role in reflecting a trainees’ commitment and professionalism as well as preserving their rights in case of any problems encountered during the program.

- 8. Role of portfolio assessment in practice

The portfolio had a positive effect on the careers of all participants, but in different ways. Some improved their clinical practice and time management in consultation particularly through Mini-CEX. Other participants found that the documentation of the cases in the portfolio helped them to remember the cases in their current clinical practice and treat the patients accordingly. One participant appreciated the benefit of portfolio assessment in teaching them how to document cases in medical records. Furthermore, some of the impact of the portfolio activity in practice are demonstrated in these quotes:

*I apply the reflection in my work, documenting some cases and their discussions along with difficulties and issues faced along the case.*

*The last time I referred to a portfolio was almost a week ago, I used it as a source. The part I most referred to is reflection.*

*Although I graduated three years ago, until now I open the portfolio* [sic].

*Searching skills that I gained in the reflection benefited me a lot in my current job, as some of the things I note it down might have changed. So, the method of looking for information benefitted me even if I didn’t go back to the portfolio itself* [sic].

- 9. Suggestions for portfolio improvement

The participants suggested some strategies to improve the outcome of portfolio assessment. Most of the suggestions concerned the implementation and process of the portfolio assessment. A need to standardise the understanding of the portfolio among all the trainees and trainers was suggested by most of the participants. Furthermore, a reduction in paperwork was suggested by most of the participants. They recommended an open structure portfolio with an open number of cases and an open deadline to decrease stress and allow a good selection of cases. Some participants suggested increasing the number of Mini-CEX and case-based discussions while others thought that less frequent use of these forms would be an advantage. An electronic portfolio was suggested by one participant who thought that writing on paper is inappropriate in view of current technological developments.

*I think that an electronic portfolio will be easier for the mentor to catch up with. Communication also will be easier this way; he could evaluate me online with no need to meet in person.*

Many suggestions concerned the use of feedback to improve portfolio assessment.

*The follow-up should be for the first two months where there is a meeting for all the trainers and trainees to discuss the achievements in some portfolios, so that the picture becomes clearer for everyone. Afterwards, each trainee can follow-up with the assigned mentor.*

*I suggest a survey targeted at those who have gone through portfolios to get a result about what was useful and what was useless.*

Although all the participants valued mentor meetings in portfolio assessment, they held conflicting views about their frequency. Most participants suggested it should be more frequent while some thought it should be less frequent, particularly at the end of the program. Designing the portfolio to be speciality specific and not to include other departments in portfolio assessment was suggested by many participants. Some also recommended a selection of skills that are closely related to family medicine practice.

### Axial coding

**Fig. 1:**
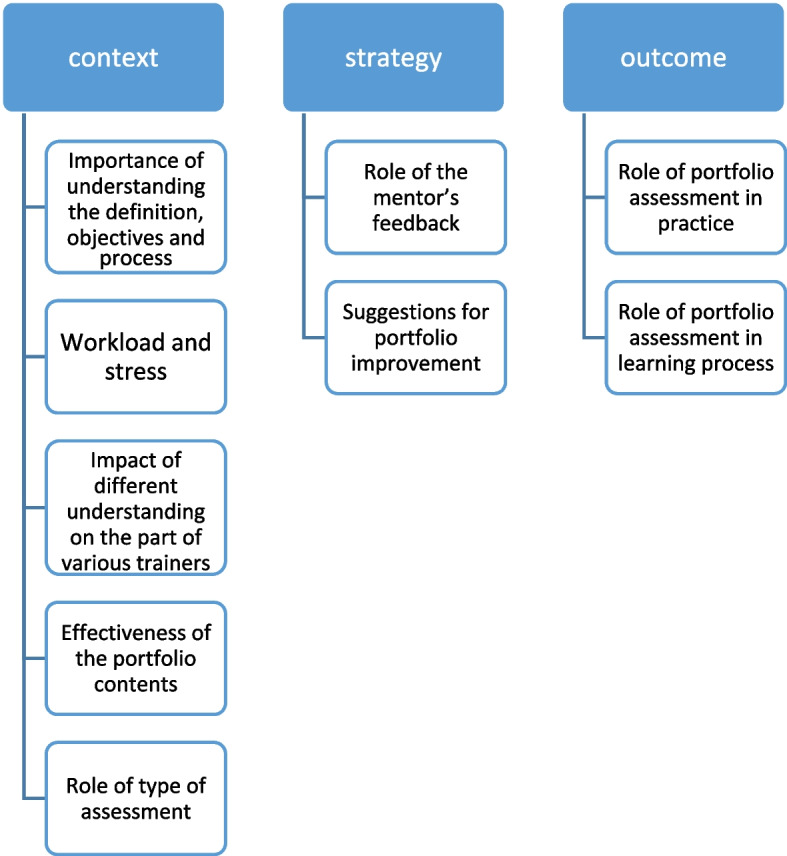
Axial coding of the open codes

In the second phase of the analysis, open codes are then regrouped according to the frequency of use of the key terms, which reflects their relevance, into three axial codes: context, strategy and outcome of portfolio assessment. Their axial relationships are illustrated in Fig. 1. Thus, our findings recognized the main characteristics which can influence the portfolio assessment; the context (what), strategy (how) and outcome (with what consequences).

## Discussion

In the training programme, the portfolio process is considered an interaction between the trainee and mentor under the umbrella of the educational system and organization. Each point of this triangle, trainee, mentor, and the system, plays a major role in the success of portfolio methodology assessment. Therefore, this study aims to explore the process of portfolio assessment by taking a broad and pragmatic look at participants’ perceptions of portfolio assessment in the SDFM programme. Data from this study showed that portfolio assessment was a useful experience among all the participants despite some challenges and difficulties, which were encountered in different aspects of portfolio methodology assessment. The participants highlighted some portfolio strengths and weaknesses and made recommendations for improvement, most of which were compatible with other evidence. The findings of the study generally support the existing literature. In medical education, assessment tools should support the learning process and measure performance simultaneously, which portfolios can do if several conditions are met. Some of these conditions are proper implementation with clear purposes and guidelines for portfolio assessment; selection of competent trainers and efficient training in courses and workshops; feedback from the trainees and trainers; and availability of support at each part of the program [20].

### Challenges

The most challenging period of the portfolio assessment was at the beginning when most of the participants were uncertain of the usefulness of the portfolio methodology. In this study, the participants’ responses to the introduction of the portfolio matched the initial negative reactions of students at the University of Dundee Medical School following the introduction of the portfolio at that institution [9]. Poor understanding of the portfolio methodology led to stress for most of the participants. One study [21], which was conducted on PGME had similar findings in which the participants reported lack of clear purpose and instruction which led to poor understanding. They recommend initiating a generic format of portfolio-based training and assessment in PGME. They also recommend orientation of the stakeholder at the beginning of portfolio utilisation.

The different understandings of portfolio assessment among the mentors in this study might not reflect a defect in the trainers’ understanding, but rather reflect weaknesses reported in some evidence about portfolio assessment, as mentioned in AMEE Guide no. 45, ‘the evidence held by a portfolio is often not standardised and its meaning often depends on the context from which it originates’ [22]. Furthermore, in one study conducted on the assessors of portfolios [23], the individuality of the portfolios and variation in starting points among different trainers were identified as the main areas of difficulty in portfolio assessment. This might indicate that the starting point of portfolio assessment might also be a difficulty for mentors.

The type of assessment in the SDFM program, either summative or formative, was vague for some participants until late in the program. This again might indicate the importance of clear purposes, guidelines, and instructions regarding portfolio assessment before implementation [14]. In fact, effective assessment in medical education is usually supported by a comprehensive grading and reporting system, which helps by clarifying expectations, maintaining a reasonable workload, and self-assessment promotion [24]. Most of the participants supported summative assessment to raise the value of the portfolio assessment. This view fits with evidence that presumes that learners will only put effort into the portfolio if it is rewarded. Grades are the most important reward in any teaching program [22]. In another study [25], mentors found that portfolios may not be taken seriously by the students or mentors if they are not included in the summative assessment. On the other hand, some participants valued the formative assessment of the portfolio as a means of support for the trainees all through the program without the stress of an exam. The remaining participants were in favour of a mixed summative and formative portfolio assessment.

### Participant perceptions of effectiveness

In this study, the participant perceptions of the effectiveness of different assessment tools were affected mainly by the number of required documents in the portfolio assessment. Reflection and Mini-CEX were reported to be the most beneficial assessment tools, but at the same time, were the least frequent requirements. This points to the importance of a reduction in paperwork to increase the effectiveness of the portfolio. However, the reflection process, and the Mini-CEX are rich in feedback, which may also play a role in trainee preferences. The participants reported a higher workload and stress when completing the logbook of clinical diaries and DOPS compared with MiniCEX. This fits with a study finding of low overall engagement of family medicine registrars in portfolio assessment, particularly in logbook and DOPS [20]. Again, the stress could be either due to workload or deficiency in the feedback.

One participant stated a preference for an electronic portfolio, which is consistent with evidence that found that the sheer bulk of paper-based portfolios is difficult for students as well as for assessors [22, 23]. Many medical schools prefer the e-portfolio as it is easier to keep up to date and hyperlinks can be inserted to connect evidence with reflection [25]. Notably, workload and stress with the portfolio was not perceived as often by participants who had experience with portfolio assessment. This fits with some evidence which reports that students with no previous experience of portfolio assessment are usually more anxious about the introduction of the portfolio methodology [26]. This fact might reflect the importance of previous exposure of undergraduate medical students to the portfolio methodology, as it will help in postgraduate education along with professional career [27].

Feedback is an important requirement for effective assessment. Mentor feedback was valued highly by all the participants. They linked the portfolio’s effectiveness in the learning process to mentor feedback. This particular benefit of the portfolio in mentor meetings was highlighted in several reports in the literature [3, 20]. The participants appreciated the educational role of the mentor’s feedback in their learning process through the identification of their learning needs, strengths, weaknesses and methods for personal and professional development and improvement, which all match the benefits reported in several studies [20, 22, 27, 28]. One of the participants’ preferred forms of documentation is the feedback on the reflection. This participant preference was reported in another study [29], in which students were encouraged to document meaningful formative feedback. Emotional support was reported as an important benefit of mentor feedback. The emotional support provided by the mentor in portfolio assessment is usually delivered through feedback on the participants’ documentation of their reflection [27, 30]. Our participants thought that the trainees who continued to be dissatisfied with portfolio assessment were not adequately instructed and followed up by their mentor. They proved this assumption by noting continued dissatisfaction among participants assigned to particular mentors. This fits with evidence which has shown that learners are usually more satisfied in working with portfolios if their mentors appreciate their efforts in portfolio assessment [22]. However, in one study [31], it was reported that learner initiative is an important factor in receiving feedback and the learner should actively seek it.

### Key findings

In this study, in which all the participants had completed the training program and were already in their work placements, all of them valued the portfolio’s impact in their practice. The effects of the portfolio were reported by the participants in different parts of portfolio assessment. Reflection was the assessment tool which most affected their practice, followed by the Mini-CEX. All of these perceptions suggest that the portfolio methodology has an important impact on professional development, which is supported by several studies [3, 6, 22, 27]. Some participants suggest a survey to evaluate the portfolio methodology of assessment for future improvement. This fits with current literature that supports the regular evaluation of the portfolio and mentoring process to ensure organisational revision and further development of mentoring competencies [32].

### Strengths

The strength of this study can be appreciated in its methodology as the results and analysis are thoroughly grounded by the data obtained and thus free from pre-existing data and knowledge. The outcomes of this study fit with much of the evidence in the existing literature which may support its possible generalizability in other contexts or settings which have a similar professional community [33].

### Limitation of this study

This study was conducted on family physicians in a particular program in one city, Riyadh. This could affect the generalizability of the outcomes to different contexts such as other postgraduate programs for other specialties, or family medicine programs in other areas of Saudi Arabia or in another country. The number of participants in the study was relatively small. However, the last interview did not provide new themes which might indicate some sort of data saturation, which cannot exclude the possibility of selection bias. The involvement of only one interviewer might be considered a limitation as well, but this would also help to ensure consistency. This study focused on participants’ perceptions, which is subjective self-reporting of the effect of the portfolio on their learning process and practice, rather than an objective measurement of the portfolio effect in their learning during the program or their practice afterwards.

### Implications for educators

This study provides some evidence that the portfolio can be a powerful tool for learning and assessment if the following recommendations are considered:

1. A manual or generic format should be created for trainees and trainers, including a clear purpose, guidelines of portfolio assessment.
2. Faculty development should be conducted for mentors and trainers who will be involved in the portfolio assessment with subsequent faculty meetings for portfolio changes and updates.
3. The assessment criteria should be defined with a clear reporting and grading system
4. An orientation course should be conducted including theoretical lectures and practical workshops in portfolio assessment for new trainees with the attendance of all mentors, trainers and some previous students who have had experience with portfolios,
5. Group feedback sessions for the trainees about portfolio assessment with attendance of the program director should be scheduled every three months for follow up of the process, ensuring group standardisation and early detection of any problems.
6. A survey should be distributed for all trainees, trainers and mentors at the end of the program for internal evaluation and future improvement.
7. The training program should be concluded by a faculty meeting to discuss the trainees’ survey and mentors’ view with flexibility of effective change implementation.
8. Consideration should be given in the future developing the program in an electronic format, which is becoming more popular and essential because of the possibility of future events such as the COVID-19 pandemic.

### Implications for future research

The findings of this study provide promising opportunities for future research. The portfolio process should be explored further, to take into consideration mentors’ perceptions to gain a good balance in understanding the methodology of portfolio assessment. Future research is needed to conduct a Kirkpatrick level 2 and 3 training evaluation of learning and behaviour of postgraduate candidates to measure its genuine effect in health care practice. Furthermore, this study can help researchers to develop a generic format of portfolio assessment through identifying the key elements of portfolio success.

## Conclusion

This study explored a general explanation of portfolio assessment shaped by the postgraduate students. It has identified the importance of understanding the portfolio methodology in assessment of skills. Thus, proper implementation is vital for the success of portfolio assessment. Paperwork and time demands were the main obstacles in portfolio assessment. The students’ perception of the reflection as the most valuable aspect which facilitated their learning, confidence, and self-assessment. Mentor feedback is a good strategy for coping with portfolio challenges. Our findings provide some evidence of positive outcomes of the portfolio methodology in practice and professional development.

## Supplementary Information

**Additional file 1.**

## Data Availability

The datasets used and/or analysed during the current study available from the corresponding author on reasonable request.
