# Supplementary material for: Perceptions of portfolio assessment in family medicine graduates: a qualitative interview study
Source: BMC Med Educ. 2022 Dec 30;22:905. doi: 10.1186/s12909-022-03991-7 (PMC9802017; doi:10.1186/s12909-022-03991-7)
Supplement: Supplementary file 1 — Additional file 1. [file 12909_2022_3991_MOESM1_ESM.docx]

**APPENDIX 1**

**(Interview Guide)**

| **Interview protocol project:** Perceptions of portfolio assessment in participants in the Saudi Diploma of Family Medicine (SDFM) programme.  **Time of Interview:**  **Date**:  **Place:** Postgraduate Centre of Family Medicine in Saudi Arabia  **Interviewer:**  **Interviewee:**  **Position of interviewee**: Participants in the Saudi Diploma of Family Medicine  This study aims to explore the process of portfolio assessment in the SDFM programme, and to develop a corresponding explanatory theory    **Questions:**   1. What do you think about portfolios in general? 2. What do you think about portfolios as part of the formative assessment process (strengths, weaknesses and challenges)? 3. What was the role or effect of the portfolio in your learning? Did it help or obstruct your achievement of learning goals? Why? 4. If you were given the chance to join another postgraduate programme in the future, would you prefer a portfolio assessment or not? Why? 5. What is the effect of portfolio assessment in your current professional career? 6. What would you suggest for the improvement of portfolios?   **Thank you for your participation in this interview. All data will be registered anonymously to keep it confidential. I will provide you with a copy of the study after finalization.** |
| --- |
